# Short Communication: A Simple Method for Performing Worm-Egg Counts on Sodium Acetate Formaldehyde-Preserved Samples

**DOI:** 10.1155/2012/617028

**Published:** 2012-12-16

**Authors:** Wayne Melrose, Holly Menzies, Melissa Boer, Hayley Joseph, David Reeve, Richard Speare

**Affiliations:** Anton Breinl Centre for Public Health and Tropical Medicine, James Cook University, Townsville, QLD 4811, Australia

## Abstract

The Kato Katz method is the most common way of performing worm-egg counts on human faecal samples, but it must be done in the field using freshly collected samples. This makes it difficult to use in remote, poorly accessible situations. This paper describes a simple method for egg counts on preserved samples collected in the field and sent to a central location for further processing.

## 1. Introduction

Faecal worm-egg counts for baseline surveys and for on-going monitoring and evaluation are an essential part of deworming programs [1]. The Kato Katz method [2, 3] is the most common way of performing worm egg counts on human faecal samples, but it requires the examination of fresh faeces in the field. That makes it very difficult to use in areas such as the Central Pacific where it may mean deploying a team of microscopists, their equipment, and supplies to a remote island only accessible by an unreliable shipping service, or the remote areas of Papua New Guinea where access is by expensive air services that can be delayed for days, and even weeks, by adverse weather. Other problems with the Kato Katz method include the need to examine the preparation within 30 minutes to prevent overclearing and underestimation of hookworm eggs [1], the inability to keep samples or slides for reexamination for quality control, the risk of infection from the faeces sample, and last but not least, the safe disposal of the discarded materials, especially glass slides, on small isolated atolls and islands where the dealing with of any sort of rubbish is difficult. 

All of the above problems can be overcome by using Sodium acetate, acetic acid, formalin (SAF)-preserved samples [2] which remain stable for long periods even when stored at ambient temperature. The collection containers and preservative can be sent to the survey site ahead of time, the samples collected by local health workers and sent to a central laboratory for processing when transport is available. The problem is devising a method for egg counts on faecal samples that have already been diluted with preservative. One way to do this is to add a weighed amount of faeces to a known volume of preservative but that would require suitable balances to be available at the field collection sites. The newly introduced “FLOTAC” method [4] can also be used on preserved samples but requires specialised equipment. Other advantages of having SAF-preserved samples is that they can be used to conduct a more comprehensive parasitological survey because concentration methods can be used to detect “rare” eggs and permanent stains can be used to detect protozoa. 

## 2. Materials and Method

SAF is added to the faecal samples as soon as possible after collection in an approximate ratio of one part of sample to two of fixative, and thoroughly homogenized using a wooden “tongue depressor” split lengthways. No lumps of faecal matter must remain as they will prevent thorough fixation of the sample. Any obvious extraneous matter such as stones and leaves is also removed at this time.

After arrival the laboratory the samples are transferred into a plastic 10 mL tube and centrifuged at a sufficient time and speed to tightly pack the faecal material into the bottom of the tube. Some experimentation will be required depending on the type of centrifuge and the density and composition of the faecal matter. In our laboratory optimal results are obtained by centrifuging at 500 g for 10 minutes. Carefully remove the supernatant leaving as little behind as possible. *Careful attention to the next step is absolutely crucial to the success of the method.* The packed faeces must be thoroughly and vigorously mixed to ensure that any worm eggs are evenly redistributed throughout the faecal material. This is best accomplished by using half of a wooden tongue depressor plus a vortex mixer.

A supply of Kato Katz templates is required. Our laboratory uses those made by Vestergaard Frandsen because they are made of plastic and can be washed and reused, but any brand will do. Place a template on to a microscope slide, fill the hole in the centre with the faecal matter, level it off, and carefully lift off the template leaving a measured amount of faecal matter (typically 41.7 mg) on the slide. Add a drop of water, mix well, and place a 22 × 22 mm cover slip on top. (Using a 22 × 40 cover slip will give a thinner preparation but will take longer to examine.) Count the eggs under the microscope, paying particular attention to the edge of the cover slip. Multiply the number of eggs counted by 24 to calculate eggs per gram (EPG).

## 3. Results and Discussion

A formal side by side comparison between the Kato Katz and this method has not been done in the field, but a laboratory-generated comparison was done by mixing a fresh, unpreserved faecal sample containing Hookworm eggs and another containing *Ascaris lumbricoides* and *Trichuris trichiura* eggs with uninfected faeces to produce samples with a range of egg counts. Kato-Katz counts were done on each of these samples after which SAF was added and the counts repeated using the new method. The comparative results are in Table 1.

Because of the small number of samples it was not possible to compare the results obtained for the three species individually, but all three were easily identified in slides prepared by the new method and the visual clarity was excellent. Hookworm eggs had disappeared completely from slides prepared by the Kato Katz method when they were reexamined next day, but were still perfectly visible in slides prepared by the new method. If the slides prepared by the new method are sealed around the edges with a hard drying mounting medium or nail varnish they will last for months, thus providing a valuable source of training materials. This is in direct contrast to the Kato-Katz preparations which cannot be kept for more than a day or two before drying out and becoming useless. 

Because this is an artificial laboratory-based experiment using a small number of “spiked” samples rather than a carefully conceived field-based trial using real participants it is difficult to draw any firm conclusions on the comparative results, but some important observations can be made. Like the Kato Katz, this method appears to be unreliable at detecting low-density infections below 100 EPG, but both methods detected similar numbers of positive samples. Very importantly, although there was a variation in egg counts between the methods, both of them correctly classified 29 out of 30 samples as representing “light intensity infections” using the World Health Organization classification [1].

One of the criticisms that could be leveled at this method is that the centrifugation artificially changes the fluid content and therefore the consistency of the sample. The Kato Katz method makes no allowance for the fluid content or consistency of the faecal sample and this has been highlighted as a potential source of error [5, 6]. It can be argued therefore that packing down the faecal matter by centrifugation will reduce this error. It must also be remembered that both methods can best described as “semiquantitative” and that extreme accuracy is not required or expected.

## 4. Conclusions

This newly described method shows great promise as a way to conduct parasite surveys and monitor deworming programs in remote, difficult to reach, and logistically challenging locations where the use of the Kato Katz method is impossible, but some field-based comparisons need to be made. 

## Figures and Tables

**Table 1:** Comparative counts in EPG.

Sample number	Kato Katz	New method
1	864	1120
2	528	938
3	408	216
4	1800	2232
5	400	284
6	6384	4938
7	3072	2088
8	280	456
9	4032	2850
10	0	72
11	2064	1700
12	120	0
13	2829	1856
14	0	128
15	0	0
16	1920	1200
17	408	827
18	120	0
19	48	0
20	360	432
